# Venous thromboembolism in non-small cell lung cancer patients who underwent surgery after induction therapy

**DOI:** 10.1007/s11748-020-01351-0

**Published:** 2020-04-09

**Authors:** Yasunori Kaminuma, Masayuki Tanahashi, Eriko Suzuki, Naoko Yoshii, Hiroshi Niwa

**Affiliations:** Division of Thoracic Surgery, Respiratory Disease Center, Seirei Mikatahara General Hospital, 3453, Mikatahara-Cho, Kita-Ku, Hamamatsu, Shizuoka 433-8558 Japan

**Keywords:** Non-small cell lung cancer, Venous thromboembolism, Pulmonary embolism, Deep vein thrombosis, Induction therapy

## Abstract

**Objectives:**

Lung cancer patients have been reported to have a high incidence of venous thromboembolism (VTE) and a high recurrence rate of VTE. However, there are no detailed reports of VTE in lung cancer patients who underwent surgery after induction therapy. We examined the incidence and clinical features of VTE in these patients.

**Methods:**

We retrospectively evaluated 89 patients with non-small cell lung cancer who underwent surgery after induction therapy at our department between April 2009 and March 2018. The incidence of VTE, clinical features, and long-term prognosis were retrospectively examined.

**Results:**

Among the 89 patients, 4 (4.5%) developed VTE, and there was no significant difference in the background characteristics between patients with and without VTE. All four patients developed VTE during preoperative treatment. In the patients with VTE, anticoagulant therapy with oral anticoagulants was administered after heparinization, and the median duration of anticoagulant therapy was 18.7 months. There were no cases of symptomatic VTE recurrence after surgery, regardless of lung cancer recurrence. Although the overall survival (OS) showed no significant difference between patients with and without VTE, the disease-free survival was significantly shorter in patients with VTE than in those without it (median 6.3 vs. 71.6 months, *p* < 0.01).

**Conclusions:**

In induction cases, the incidence of VTE was 4.5%, and it can at least be stated that no symptomatic VTE developed or recurred after surgery. Patients with VTE in induction therapy had short progression-free survival and required careful follow-up after surgery.

## Introduction

Venous thromboembolism (VTE) consists of pulmonary thromboembolism (PE) and deep vein thrombosis (DVT). VTE occurs in 4–20% of cancer patients, and lung cancer is reported to carry a high risk of VTE complications among solid tumors [1–3]. Cancer patients also have a high VTE recurrence rate [4]. Thrombus formation is promoted as follows: coagulation factors are activated by tissue factors and procoagulants secreted from cancer cells, and vascular endothelial cells are damaged by mucin-like substances and cytokines derived from cancer cells [1]. Patients with unresectable/recurrent cancer are at a high risk of VTE recurrence, due to the persistent hypercoagulable state caused by cancer. However, if a cancer-free status can be obtained by surgery after induction treatment for advanced lung cancer, the internal environment surrounding VTE may differ from that in patients with unresectable/recurrent cancer. In this study, we clarified the incidence rate, high incidence period, and recurrence rate of VTE in patients who received induction therapy, and examined the long-term prognosis of these patients.

## Patients and methods

**Fig. 1:**
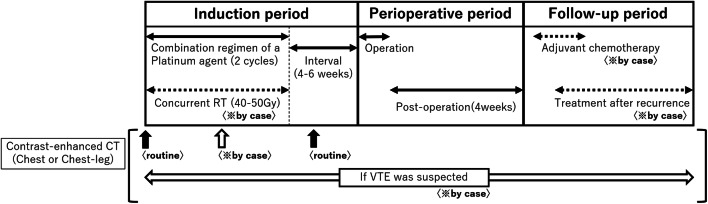
Diagram for the treatment and contrast enhanced-CT (chest or chest–leg) follow-up. The treatment phase was divided into induction, perioperative, and follow-up periods. We examined in which periods VTE was more likely to develop. Routine contrast enhanced-CT follow-up was performed before induction therapy and when assessing the therapeutic effects of induction therapy. In other cases, we performed contrast-enhanced CT when assessing the therapeutic effects at the midpoint of induction therapy and when symptomatic VTE was clinically suspected

We included 89 patients with non-small cell lung cancer (NSCLC) who underwent surgery after induction therapy in our department from April 2009 to March 2019. The incidence of VTE, clinical background characteristics, and the recurrence rate were examined retrospectively. We divided the treatment phase into induction, perioperative, and follow-up periods, and examined which periods were more likely to develop VTE (Fig. 1). The inclusion criteria for induction therapy were patients with mediastinal lymph node metastasis (N2 or 3) and/or direct invasion of nearby organs (T3 or 4), up to 70–75 years old, and an Eastern Cooperative Oncology Group performance status (ECOG-PS) of 0. The neoadjuvant chemotherapy regimen was platinum-based doublet chemotherapy, and radiation therapy with 40–50 Gy was performed whenever possible. Before the start of treatment, contrast-enhanced computed tomography (CT), positron emission tomography (PET)-CT, and brain magnetic resonance imaging (MRI) were performed, and all patients were histologically diagnosed with NSCLC. In patients for whom lymph node metastasis was suspected, a histological evaluation was performed with endobronchial ultrasound- guided transbronchial needle aspiration (EBUS-TBNA) or mediastinoscopy whenever possible. The 8th edition of the TNM classification was used to determine the lung cancer staging, and the therapeutic effect was judged according to the Response Evaluation Criteria in Solid Tumors version 1.1 (RESIST v.1.1).

Contrast-enhanced CT was performed in all cases when clinical symptoms suggestive of VTE appear during treatment and when determining the effect of preoperative therapy.

Regardless of the presence or absence of symptoms, anticoagulation with unfractionated heparin (UFH) was immediately performed when VTE was detected on contrast-enhanced CT. After that, the medication was changed to an oral anticoagulant and continued to be taken until the patient’s condition stabilized. Exclusion criteria were patients in whom the absence of VTE could not be confirmed before the start of induction therapy, those who started preoperative treatment but for whom surgery was deemed difficult, and those in whom contrast CT was deemed difficult. We also excluded patients with non-controllable infections, diabetes, hypertension, and severe pre-existing conditions, including double cancer in need of treatment.

This study was approved by the ethics committee of our institution (authorization number 19–20). The requirement of informed consent from each patient was waived due to the retrospective study of the data.

### Statistical analyses

Regarding the statistical methods, Fisher's exact test and the Mann–Whitney *U* test were used for background comparisons. The survival curve was calculated by the Kaplan–Meier method. The log-rank test was used for the significance test, and significant differences were determined at *p* < 0.05. All statistical analyses were performed with R (The R Foundation for Statistical Computing, Vienna, Austria) and EZR (Saitama Medical Center, Jichi Medical University, Saitama, Japan), which is a modified version of R commander designed to add statistical functions frequently used in biostatistics [5].

## Results

### Patient characteristics

Of the 89 patients undergoing induction therapy, 4 (4.5%) developed VTE. There was no apparent significant difference in the background of patients with and without VTE (Table 1). The smoking history, stage, histological type and pathological therapeutic response varied among patients with VTE. However, the one thing that they all had in common was the use of cisplatin-based chemotherapy and radiation combination therapy. (Table 2).

**Table 1:** Characteristics of patients with and without VTE undergoing induction therapy followed by surgery

	Total *n* = 89	Patients with VTE *n* = 4	Patients without VTE *n* = 85	*p* value
Sex				0.502
Male	75 (84.3%)	3 (75.0%)	72 (84.7%)	
Female	14 (15.7%)	1 (25.0%)	13 (15.3%)	
Age				0.186
Median (range)	62.0 (36–73)	60.5 (52–71)	59.9 (36–73)	
ECOG PS				1
0	89 (100%)	4 (100%)	85 (100%)	
Smoking status				0.319
Never smoker	8 (9.0%)	1 (25.0%)	7 (8.2%)	
Former or current	81 (91.0%)	3 (75.0%)	78 (91.8%)	
Smoking index (pack years)				0.324
Median (range)	37.4 (0–106)	48.3 (0–106)	36.9 (0–82)	
CT total tumor size				0.804
Median (range)	5.1 (1.1–30)	4.6 (1.7–10.0)	5.1 (1.1–30)	
PET-CT SUVmax				0.376
Median (range)	12.2 (2.8–25.0)	14.4 (2.8–22.0)	12.1 (3.3–25.0)	
Histology				0.186
Squamous	30 (33.7%)	2 (50.0%)	28 (32.9%)	
Adenocarcinoma	49 (55.1%)	1 (25.0%)	48 (56.5%)	
Adenosquamous	3 (3.4%)	1 (25.0%)	2 (2.4%)	
Pleomorphic carcinoma	3 (3.4%)	0 (0.0%)	3 (3.5%)	
NSCLC	4 (4.5%)	0 (0.0%)	4 (4.7%)	
Clinical T factor				0.09
T1	15 (16.9%)	2 (50.0%)	13 (15.3%)	
T2	15 (16.9%)	0 (0.0%)	15 (17.6%)	
T3	29 (32.6%)	2 (50.0%)	27 (31.8%)	
T4	30 (33.7%)	0 (0.0%)	30 (35.3%)	
Cinical N factor				1
N0	30 (33.7%)	2 (50.0%)	28 (32.9%)	
N1	11 (12.4%)	0 (0.0%)	11 (12.9%)	
N2	42 (47.2%)	2 (50.0%)	40 (47.1%)	
N3	6 (6.7%)	0 (0.0%)	6 (7.1%)	
Clinical stage				0.543
IIB	22 (24.7%)	2 (50.0%)	20 (23.5%)	
IIIA	47 (52.8%)	2 (50.0%)	45 (52.9%)	
IIIB	19 (21.4%)	0 (0.0%)	19 (22.4%)	
IIIC	1 (1.1%)	0 (0.0%)	1 (1.2%)	
Regimen of neoadjuvant therapy				1
CDDP + DTX	40 (44.9%)	2 (50.0%)	38 (44.7%)	
CDDP + VNR	36 (40.5%)	2 (50.0%)	34 (40.0%)	
CBDCA + PTX	11 (12.4%)	0 (0.0%)	11 (12.9%)	
CBDCA + DTX	2 (2.3%)	0 (0.0%)	2 (2.4%)	
Radiation dose of neoadjuvant therapy				0.426
40 Gy	32 (36.0%)	3 (75.0%)	29 (34.1%)	
50 Gy	47 (52.8%)	1 (25.0%)	46 (54.1%)	
None	10 (11.2%)	0 (0.0%)	10 (11.8%)	
RECIST tumor response				1
Complete response	1 (1.1%)	0 (0.0%)	1 (1.2%)	
Partial response	46 (51.7%)	2 (50.0%)	44 (51.8%)	
Stable disease	41 (46.1%)	2 (50.0%)	39 (45.9%)	
Progressive disease	1 (1.1%)	0 (0.0%)	1 (1.2%)	
Pathological therapeutic response				0.838
Ineffective (Ef.0)	1 (1.1%)	0 (0.0%)	1 (1.2%)	
Slightly effective (Ef.1)	35 (39.3%)	1 (25.0%)	34 (40.0%)	
Moderately effective (Ef.2)	37 (41.6%)	2 (50.0%)	35 (41.2%)	
Markedly effective (Ef.3)	16 (18.0%)	1 (25.0%)	15 (17.6%)	
Complete resection	80 (89.9%)	4 (100.0%)	76 (89.4%)	1
Incomplete resection	9 (10.1%)	0 (0.0%)	9 (10.6%)	
No recurrent survival	38 (42.7%)	2 (50.0%)	36 (42.4%)	1
Recurrent survival	15 (16.9%)	0 (0.0%)	15 (17.6%)	
Death	36 (41.5%)	2 (50.0%)	34 (40.0%)	
Cancer death	24	2	22	

Pathological therapeutic response: Ef 0, no therapeutic effect; Ef 1a, residual viable cancer cells detected in “≥ 2/3” of resected tumor; Ef 1b, residual viable cancer cells detected in “< 2/3 and ≥ 1/3” of resected tumor; Ef 2, residual viable cancer cells detected in “< 1/3” of resected tumor; Ef 3, no residual viable cancer cells [14]

*PET-CT* positron emission tomography-computed tomography, *SUVmax* maximum standardized uptake value, *NSCLC* non-small cell lung cancer, *CDDP* cisplatin, *DTX* docetaxel, *VNR* vinorelbine, *CBDCA* carboplatin, *PTX* paclitaxel, *VTE* venous thromboembolism

**Table 2:** Clinical background characteristics and treatment of patients with VTE

Patient no	Age	Sex	Smoking status (pack year)	Primary lesion	Histology	Onset of VTE from starting ICRT (day)	Treatment phase at onset of VTE	State of VTE	Symptoms at onset	Clinical TNM	ICRT regimen	Concurrent RT dose
1	71	M	51	RUL	Sq	56	Induction phase	PE + DVT	Asymptomatic	T3N0M0	CDDP + VNR	40 Gy
2	62	F	0	RUL	Adsq	51	Induction phase	PE + DVT	Asymptomatic	T3N0M0	CDDP + VNR	40 Gy
3	52	M	106	LUL	Ad	43	Induction phase	PE + DVT	Asymptomatic	T1aN2M0	CDDP + DTX	50 Gy
4	57	M	36	RUL	Sq	24	Induction phase	PE + DVT	Asymptomatic	T1cN2M0	CDDP + DTX	40 Gy

Patient no.	Therapeutic response (RECIST v.1.1)	Pathological therapeutic response	Adjuvant chemotherapy	Anticoagulant agent (after heparinization)	Period of anticoagulation (month)	Outcome	Recurrent site	Recurrence of VTE
1	SD	Moderately (Ef.2)	No	Warfarin	13.6	Cancer death	Brain	No
2	PR	Moderately (Ef.2)	No	Warfarin	24.9	Cancer death	Brain, skin	No
3	SD	Slightly (Ef.1b)	CBDCA + PTX	Edoxaban	23.7	Recurrent survival	Adrenal grand	No
4	PR	Markedly (Ef.3)	CDDP + DTX	Edoxaban	6.2	Recurrent survival	Lung (bilateral), bone	No

All patients with VTE had PE and DVT, but they were asymptomatic. In all cases, VTE was diagnosed accidentally by contrast-enhanced CT performed during and at the end of induction therapy. The median period of VTE onset from the start of induction therapy was 47 days (24–56 days), and all patients developed VTE during the induction period. In patients with VTE, after the administration of UFH, oral anticoagulants (Warfarin/Edoxaban = 2/2 cases) were continuously administered for a median of 18.7 months (6.2–24.9 months). The anticoagulant was discontinued before the operation and resumed early after the operation. There were no adverse events associated with anticoagulant therapy, including bleeding, and there was no recurrence of VTE after discontinuation.

### The prognosis

**Fig. 2:**
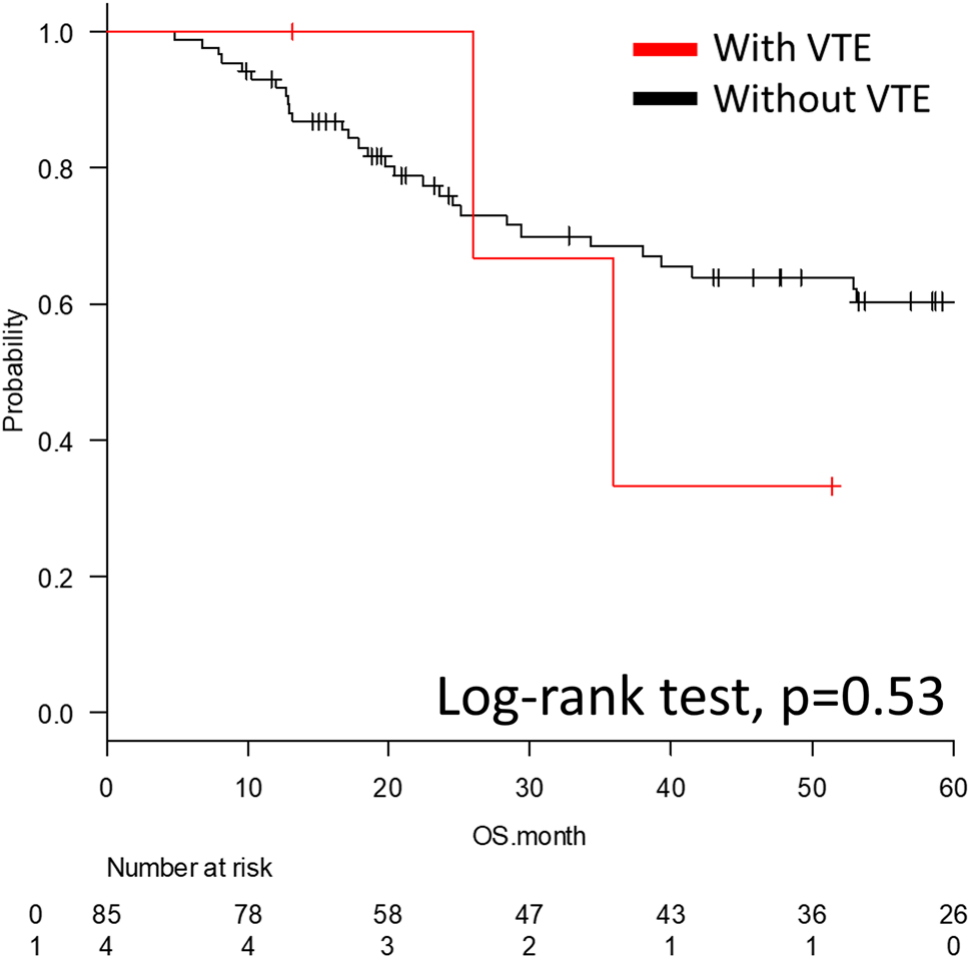
Kaplan–Meier curve of the 5-year overall survival for the patients with and without venous thromboembolism

There was no significant difference in the 5-year overall survival (OS) when comparing the prognosis of the patients with and without VTE (log-rank test, *p* = 0.53). The 5-year OS rate of the patients with VTE was not reached, and the median OS was 35.9 months (95% confidence interval [Cl] 26.0–N/A months). The 5-year OS rate of the patients without VTE was 60.3%, and the median OS was 83.3 months (95% Cl 52.9–N/A months) (Fig. 2).

**Fig. 3:**
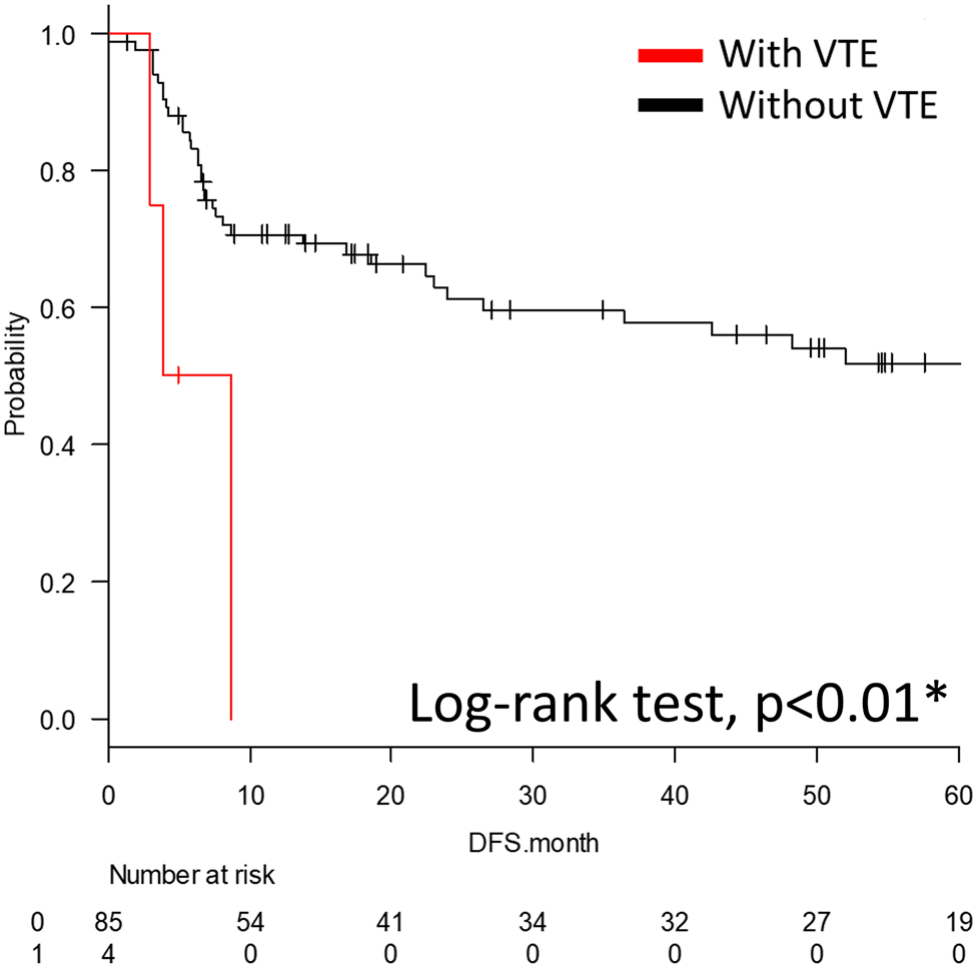
Kaplan–Meier curve of the 5-year disease-free survival for the patients with and without venous thromboembolism

There was a significant difference in the 5-year disease-free survival (DFS) when comparing the prognosis of the patients with and without VTE, with the patients with VTE showing a significantly worse prognosis (log-rank test, *p* < 0.01). The 5-year DFS rate of the patient with VTE was 0%, and the median DFS was 6.3 months (95% Cl 2.9–N/A months). The 5-year DFS rate of the patients without VTE was 51.9%, and the median DFS was 71.6 months (95% Cl 23.9–N/A months) (Fig. 3).

Prognostic factors in induction therapy cases were examined by a univariate analysis, and there were no significant prognostic factors for the OS. In DFS, the presence of VTE and a poor pathological therapeutic response (Ef.0/1) were significant prognostic factors (*p* = 0.02 and < 0.01) (Table 3).

**Table 3:** Results of a univariate analysis of the predictive factors for the 5-year overall and disease-free survival

	Overall survival *p*	Disease-free survival *p*
Sex	0.61	0.17
Age (over 65 years)	0.87	0.89
Smoking (never- vs. others)	0.96	0.86
Histology (adenocarcinoma vs. others)	0.05	0.90
cStage (II vs. III)	0.32	0.80
RT (0 Gy vs. 40–50 Gy)	0.75	0.77
RECIST (CR/PR vs. SD/PD)	0.52	0.11
Complete resection	0.10	0.09
Pathological therapeutic response (Ef.2/3 or less)	0.23	0.02
VTE event	0.53	< 0.01

*Significant difference at a probability level of 0.05

## Discussion

The general incidence of VTE is reported to be 4–20% in cancer patients [1–3]. In the present study, the frequency was equivalent to 4.5% in lung cancer patients who underwent surgery after induction therapy. In a trial related to induction therapy for lung cancer, VTE was reported as an adverse event in 4% of cases. Given the above, the incidence of VTE in patients who undergo surgery after induction therapy is expected to be around 4–5% [6]. Chemotherapy with cisplatin has been reported to carry a significantly higher incidence of VTE than other platinum-based chemotherapy regimens, and all VTE cases were in the cisplatin combination group in the present study [7]. In addition, the recurrence rate of PE was reportedly significantly higher and the risk of cerebral hemorrhaging higher in cases in which chemoradiation therapy was performed than in others. However, PE recurrence and bleeding events were not observed in the present study [8]. In the studies of non-induction therapy, a dissertation reported the postoperative rate of VTE changes depending on the extent of operation (lobectomy or bilobectomy vs. pneumonectomy) and surgical approach (video-assisted thoracic surgery vs. thoracotomy) [9]. However, another study reported that the incidence of VTE did not differ according to the surgical approach (minimally invasive surgery vs. open surgery) or the extent of operation (lobectomy, wedge resection and pneumonectomy) [10]. There was no fixed consensus on the relationship between the incidence of postoperative VTE and the surgical procedures. In induction therapy for lung cancer, the causal relationship between the surgical procedures and the incidence of VTE remains unclear, because there were no postoperative cases of VTE in our study and no reports have examined the incidence of VTE.

All VTE cases in our study were asymptomatic. Anticoagulant therapy is required, regardless of symptoms, as there have been reports that anticoagulant therapy for asymptomatic DVT significantly reduces the appearance and recurrence of symptomatic VTE in patients with cancer [11]. European and American guidelines recommend low-molecular-weight heparin (LMWH) for VTE patients with cancer, but LMWH is not available in Japan. Therefore, after treatment with UFH, anticoagulant therapy with oral coagulants (Warfarin) is often continued. However, direct oral anticoagulants (DOACs) have recently been reported to prevent recurrence, and DOACs are commonly used because of their low risk of bleeding and drug interaction [12]. There is no fixed view concerning the duration of treatment, but in recent years, anticoagulant therapy for half a year or more has been recommended. The present study included cases for which the guidelines and consensus were unclear. These patients were therefore given warfarin after the administration of UFH for about 2 years (cases 1, 2). Edoxaban has recently been used as a DOAC, with anticoagulant therapy performed for half a year (cases 3, 4). Patients with unresectable/relapsed cancer have been reported to have a 20.7% risk of VTE recurrence, even if anticoagulant therapy is used [4]. In the present study, all patients developed VTE during the induction period, but had no recurrence after surgery or discontinuation of anticoagulation therapy, so anticoagulation therapy beyond the recommended period is considered unnecessary.

The DFS was significantly shorter in patients with VTE than in those without VTE (median 6.3 vs. 71.6 months, *p* < 0.01), and a univariate analysis revealed that the presence of VTE was a poor prognostic factor for the DFS. The short progression-free survival in patients with VTE might have been due to the interaction between thrombi and circulating microcancer in the blood. Substances such as tumor-derived mucin and cytokines damage the vascular epithelium and promote the formation of microthrombi in blood vessels. The microthrombi can trap cancer cells that circulate in the blood and thus function as a scaffold for metastasis [13]. Metastasis, especially distant metastasis, was considered more likely to occur in patients with VTE than in those without it, because metastasis of circulating microcancer in the blood is likely to occur due to the effect of the microthrombi.

The VTE recurrence rate of cancer-bearing patients is reportedly about 30%, but there were no case of recurrence of symptomatic VTE in this study [4]. This suggests that the disease state in the patients who develop VTE during induction therapy may differ from that in patients with unresectable/recurrent cancer. Once lung cancer is removed via surgery after induction therapy, patients become cancer free, thereby resetting their abnormalities in the coagulation system. Recurrence of VTE is thus expected to be less frequent in induction therapy cases than in unresectable/recurrent cases. However, this is only a hypothesis, and precisely why VTE does not develop after recurrence is unclear at present.

The median recurrence period for VTE cases was 6.3 months, and careful follow-up is necessary from the early postoperative period. VTE patients are reported to have a poor prognosis, but the 5-year OS was similar between patients with and without VTE in this study [3].

This study is limited by its retrospective nature, and the fact that there were only 89 patients and 4 patients with VTE, a small number. Verification in a large, prospective study is therefore necessary. Another major limitation of this study was the inability to make a direct comparison with patients with unresectable/recurrent cancer or patients who had undergone non-surgical treatment, such as radical chemoradiation therapy. In addition, regular enhanced CT and coagulation tests were not performed. Asymptomatic VTE cases were therefore not diagnosed, while contrast-enhanced CT is actively performed for symptomatic VTE. The actual onset rate of total VTE may be higher than that depicted in this study.

## Conclusion

This study is the first report investigating the incidence and long-term prognosis of VTE in lung cancer patients who underwent surgery after induction therapy. In this study, the incidence of VTE in a small group of 89 patients was 4.5%. It can at least be stated that no “symptomatic” VTE developed or recurred after surgery. DFS may be significantly shorter in patients who develop VTE during induction therapy. Careful follow-up is necessary, as cancer recurrence is seen early after surgery in patients with VTE.
